# Coronavirus Disease 2019 Diagnostics: Key to Africa's Recovery

**DOI:** 10.1089/dna.2021.0540

**Published:** 2022-01-12

**Authors:** Sanushka Naidoo, Jesse Gitaka, Sara Suliman, Sara Baptista, Blessing Mbabie Oyedemi, Emmanuel Nepolo, Shymaa Enany

**Affiliations:** ^1^ Next Einstein Forum Community of Scientists, Kigali, Rwanda.; ^2^ Department of Biochemistry, Genetics and Microbiology, Forestry and Agricultural Biotechnology Institute (FABI), University of Pretoria, Pretoria, South Africa.; ^3^ Directorate of Research and Innovation, School of Medicine, Mount Kenya University, Thika, Kenya.; ^4^ Division of Rheumatology, Inflammation and Immunity, Brigham and Women's Hospital, Harvard Medical School, Boston, Massachusetts, USA.; ^5^ Instituto de Medicina Molecular, Faculdade de Medicina, Universidade de Lisboa, Lisboa, Portugal.; ^6^ Department of Biotechnology, Michael Okpara University of Agriculture, Umudike, Nigeria.; ^7^ Department of Human, Biological & Translational Medical Sciences, School of Medicine, University of Namibia, Windhoek, Namibia.; ^8^ Department of Microbiology and Immunology, Faculty of Pharmacy, Suez Canal University, Ismailia, Egypt.

**Keywords:** COVID-19, SARS-CoV-2, diagnosis, RT-PCR

## Abstract

With the coronavirus disease of 2019 (COVID-19) becoming a full-blown outbreak in Africa, coupled with many other challenges faced on the African continent, it is apparent that Africa continues to need diagnostics to enable case identification and recovery to this and future challenges. With the slow vaccination rates across the continent, reliable diagnostic tests will be in demand, likely for years to come. Thus, access to reliable diagnostic tools to detect the severe acute respiratory syndrome of the coronavirus-2 (SARS-CoV-2), the virus responsible for COVID-19, remain a critical pillar to monitor and contain new waves of COVID-19. Increasing the local capacity to manufacture and roll-out vaccines and decentralized COVID-19 testing are paramount for fighting the pandemic in Africa.

The global weight of infectious diseases is heavier in Africa compared with other continents. According to the World Health Organization (WHO), about 50% of mortality rates in children under 5 due to measles, diarrhea, malaria, HIV, tuberculosis (TB), and pneumonia are in Africa (WHO, 2021). Similar mortality rates are also noted in African adults, with an estimated 1.6 million annual deaths due to TB, HIV, and malaria. Nonetheless, despite the escalating threat of Coronavirus Disease of 2019 (COVID-19), Africa has initially faced a relatively low fraction of global COVID-19 mortalities (Dong *et al.*, 2020). Data from 2020, generated by the United Nations showed that Africa has 1.5% of the world's reported cases of COVID-19 and less than 0.1% of the world's deaths. This is largely due to the lockdown measures implemented early on by governments (Outlook, 2020). To respond to COVID-19, many African Union member states have been using a combination of containment and mitigation measures to delay a surge in cases that could overwhelm the availability of hospital beds, while protecting the medically vulnerable, such as the elderly and those with comorbidities (Ondoa *et al.*, 2020). However, the numbers are likely underestimated due to limited testing. Specialized laboratory facilities with skilled staff and expensive equipment to undertake these tests are scarce in most African countries. Centralized laboratories with testing facilities require samples to be transported and consistent access to electricity, and systems to communicate the test results back to patients. Thus, turnaround times may be suboptimal, resulting in loss to follow-up, and a breakdown in implementing the necessary isolation and contact tracing to contain outbreaks. Point-of-care (PoC) or near-patient solutions, such as rapid antigen tests, are preferable (WHO, 2020b; trials, 2020).

In general, the ability of any country to effectively contain the COVID-19 pandemic depends on the capacity for widespread testing, early diagnosis, tracking, and contact tracing. However, there are different modalities of COVID-19 diagnositcs, where the applications are often misunderstood by the public (Page *et al.*, 2020). The current gold standard of testing for severe acute respiratory syndrome of the coronavirus-2 (SARS-CoV-2) infection is reverse transcription/polymerase chain reaction (RT-PCR), which relies on direct detection of the viral genetic material in a sample from the resipatory tract (Hellou *et al.*, 2020). This sensitive test is resource intensive and becoming increasingly inaccessible for most African countries, particularly in rural settings. For one to run a batch test for SARS-CoV-2, a laboratory facility with a biosafely level 2 rating is required with skilled staff and expensive specialized equipment.

Furthermore, completing a COVID-19 RT-PCR test is a multistep process that begins with sample collection, often at a PoC that is far away from the country's test center(s) at central laboratories. The sample must then be carefully packaged and transported, under a cold chain, to the distant centralized testing site under conditions that protect sample integrity, and reduce risk of exposure. At the laboratory, arduous sample preparation is required, including RNA extraction for most RT-PCR platforms, followed by reaction mix preparation and RT-PCR (Hellou *et al.*, 2020).

The multistep, labor-intensive RT-PCR process results in huge backlogs, severely slowing testing of suspected positive cases, and limiting capacity for mass testing. Not only that, but this tedious process poses a serious threat to undo the progress made by African countries in reducing the risk of transmission, possibly contributing to the current undersestimation of the true burden of the disease, which is revealed by SARS-CoV-2 seroprevalence studies, that is, studies measuring antibodies against the virus, which indicate prior infection (Arora *et al.*, 2021). In fact, RT-PCR diagnostic capacity is severely limited in most African countries, with the number of qualified testing laboratories ranging between 1 and 3 in 40 African countries as of mid April 2020.

There are, however, ongoing efforts to improve testing capacity in several countries, such as Kenya (Kobia, 2020). Nevertheless, the level of testing in Africa varies as a function of the economic status of the countries among other factors such as political leadership, socioeconomic realities, and stage of the pandemic. For example, South Africa, the second largest economy on the continent, adopted a mass screening strategy for SARS-CoV-2 in contrast to other countries such as Uganda, which adopted a targeted screening strategy focusing on a risk group (IPMU, 2020).

These factors, and the need for highly trained personnel, limit the capacity of African countries to run COVID-19 tests, further tipping the global imbalance against Africa. African nations' preparedness and competence in contact-tracing capacity during disease outbreaks is commendable. However, Africa's Achilles heel remains the limited access to diagnostics, particularly locally manufactured RT-PCR-based tests. Alternative SARS-CoV-2 tests that do not require nucleic acid extraction, such as rapid antigen diagnostics for PoC testing, exist (Muthamia *et al.*, 2021). Other SARS-CoV-2 tests such as near-patient solutions are preferred and one such test is a PoC RT-PCR-based Xpert Xpress SARS-CoV-2 testing with a fast turnaround (45 min) using small GeneXpert machines (Cepheid, Sunnyvale, CA). The GeneXpert platform, already in place for TB testing across most African countries, allows the use of SARS-CoV-2 cartridges, however, drawbacks include cost and scarcity of cartriges, as well as competition with TB diagnosis (Nagura-Ikeda, 2020).

The major challenges of deploying alternative testing modalities include test reliability (sensitivity and specificity), supply chain, and fitness for utility. It is anticipated that in the coming 3–6 months there will be several reliable antibody-based tests in use in Africa. However, antibody-based serological tests only indicate prior exposure to SARS-CoV-2, and do not diagnose current infection and transmission.

Rapid antigen tests are scalable and affordable alternatives to RT-PCR. Thus, they allow for decentralized testing in rural and resource-constrained communities. However, as new waves of COVID-19 ravage the globe, the reliance on supply chain from non-African developers needs to be urgently replaced with local development and manufacturing of these rapid tests.

Meanwhile, global distribution of an efficacious vaccine against SARS-CoV-2 is likely to be the most effective strategy to curb transmission of the virus. At present, the progress in deploying antiviral drugs to treat SARS-CoV-2 infection remains slow and inadequate. In contrast, there are currently over 100 vaccine candidates at different phases of development and testing globally (WHO, 2020a) with at least 10 in active clinical trials past phase 0 (Mullard, 2020), and many already licensed and deployed.

As an imperative and at each stage of these clinical trials, the safety of the vaccine is assessed by monitoring the rates of adverse events, that is, dangerous side effects of the vaccines, while the efficacy is determined usually by the rates of moderate and severe COVID-19 disease. The first stage (phase I), also known as “first-in-human” trials, aims to test safety, and may include basic immunological assays to test whether a vaccine induces a likely protective immune response. These assays are based on the most up-to-date understanding of what a protective response may look like. It is important, however, to emphasize that the collective understanding of correlates of protective immunity against COVID-19 is still an emerging and evolving field. Importantly, this phase does not test whether the vaccine is efficacious, that is, whether the vaccine “works.” The second phase usually expands on the first phase, and may include additional metrics such as determining the most effective dose of the vaccine.

Efficacy of the vaccine is only tested in phase IIb or phase III, which usually extends to multiple sites, and tests whether the vaccine can meaningfully prevent SARS-CoV-2 infections and/or severe COVID-19 disease. In efficacy trials, uninfected healthy volunteers are randomized into a control group that does not receive the vaccine, and a parallel experimental group that receives it. For a vaccine to be deemed efficacious, the rate of infections in the vaccinated arm of the trial has to be significantly lower than the control group in the same population and setting. Here, reliable diagnostics to diagnose COVID-19 are critical to definitively determine the rates of SARS-CoV-2 infection, which necessitate serial testing during the course of the trial to subsequently calculate vaccine efficacy. These trials often require enrollment of hundreds or thousands of volunteers, and have to take into account population differences, which may influence vaccine efficacy.

Increasing the local manufacturing capacity of COVID-19 vaccines is contingent on technology transfer from vaccine developers to vaccine manufacturing plants in Africa. The Ad26.COV2.S vaccine by Johnson & Johnson is already being produced in Africa. Furthermore, on July 21st, 2021, Pfizer/BioNTech announced that it will share the ingredients for its vaccine for mass production in the Biovac institute in Cape Town (Meldrum and Petesch, 2021). However, to avoid the current situation of vaccine hoarding by high-income countries, the ultimate goal is to ramp up the investment for local innovation, so that Africa leads its own vaccine development and production.

Parallel clinical trials to test therapeutics against SARS-CoV-2 have also taken place. In Africa, few countries have participated in a COVID-19 clinical trial called “The Solidarity Trial” for potential antiviral therapies launched by the WHO and partners (WHO, 2020b). This international clinical trial compared four therapeutics against standard of care to assess their effectiveness against COVID-19. The observed limited participation by African countries may have been caused by the reluctance of governments to facilitate national trial participation, due to lack of biomedical infrastrucuture and local capacity to provide regulatory vigilance and better governance of clinical trials to protect clinical trial participants. As of June 24, 2020, the first Africa assessment of COVID-19 vaccine candidate began in South Africa since South Africa has the best developed medical infrastructure in Africa (Clinical Trials Arena). Therefore, data from non-African sites may not always be generalizable to African populations, and hence, trials in other African countries are needed.

With all these factors combined, the true extent of the spread of COVID-19 in the continent is unclear and very hard to predict, raising the risk of undetected community spread (Dong *et al.*, 2020; Arora *et al.*, 2021). Furthermore, government responses to COVID-19 that are blinded to the true pandemic realities on the ground means that they may not be suitable or sufficient. For instance, measures may not have been sufficiently strong to reflect the actual prevalence and position of communities on the epidemic curve. Likewise, measures may be too stringent in some contexts if identical risk is assumed countrywide in the absence of mass surveillance data. Hence, limited diagnostic capacity confounds candid evaluations of the governments' COVID-19 responses, thus limiting the countries' abilities to deploy limited resources to maximum effectiveness.

Importantly, SARS-CoV-2 diagnostics need to be affordable and widely accessible to test vaccine efficacy in Africa, especially if vaccine trials are conducted in African communities. For a population density equivalent to almost 20% of the world population, only 44 out of the 47 countries in the WHO African region have at least one certified laboratory for SARS-CoV-2 testing (at the start of the outbreak only two could do so). Nevertheless, due to the centralized nature of these tests, only a small percentage of the population has been tested. According to the CDC, this is just over 0.1% of the African population, in stark contrast to the Organization for Economic Co-operation and Development (OECD) countries who on average test 2.3% of their population. (https://africacdc.org/covid-19/). Global supply chain constraints remain. The standard RT-PCR test kits are expensive, making the cost of large-scale testing prohibitive for African countries.

As African countries struggle to combat many infectious diseases, COVID-19 has overwhelmed global health systems, including those of developed countries. In light of the global performance surrounding COVID-19, better local solutions depend on local scientific solutions. Africa will prevail against the pandemic when the local science and research infrastructures can directly inform health care practices and policies guiding control of infectious diseases outbreaks. This should go hand in hand with increased procurement and local manufacturing of diagnostic tests for COVID-19 to allow for early case detection, contact tracing, and isolation.

The International Monetary Fund (IMF) projects that the global economy has declined by around three percent in 2020 (Outlook, 2020). However, this should not hinder African countries from strategically investing in science and innovation, which will foster local economic growth. The African Academy of Science, The African Institute for Mathematical Sciences, and The Next Einstein Forum initiative, are examples of several pan-African networks of excellence in training and research, with a community of scientists pushing forward several efforts in the global fight against COVID-19.

Yet, funding is required to support African research institutes and science initiatives that are currently developing in-house SARS-CoV-2 diagnostic tests, with the same accuracy, reliability, and convenience, at a lower fraction of the cost, in partnership with large international biopharma availability of rapid local diagnosis will help to better gauge the scale of infections and to respond accurately to pandemics, both now and in the future. It will help Africa tackle many problems at once, both at the health and economic levels.
